# CAF-1-induced oligomerization of histones H3/H4 and mutually exclusive interactions with Asf1 guide H3/H4 transitions among histone chaperones and DNA

**DOI:** 10.1093/nar/gks906

**Published:** 2012-10-02

**Authors:** Wallace H. Liu, Sarah C. Roemer, Alex M. Port, Mair E. A. Churchill

**Affiliations:** Department of Pharmacology, University of Colorado School of Medicine, Mail Stop 8303, PO Box 6511, Aurora, CO 80045, USA

## Abstract

Anti-silencing function 1 (Asf1) and Chromatin Assembly Factor 1 (CAF-1) chaperone histones H3/H4 during the assembly of nucleosomes on newly replicated DNA. To understand the mechanism of histone H3/H4 transfer among Asf1, CAF-1 and DNA from a thermodynamic perspective, we developed and employed biophysical approaches using full-length proteins in the budding yeast system. We find that the C-terminal tail of Asf1 enhances the interaction of Asf1 with CAF-1. Surprisingly, although H3/H4 also enhances the interaction of Asf1 with the CAF-1 subunit Cac2, H3/H4 forms a tight complex with CAF-1 exclusive of Asf1, with an affinity weaker than Asf1–H3/H4 or H3/H4–DNA interactions. Unlike Asf1, monomeric CAF-1 binds to multiple H3/H4 dimers, which ultimately promotes the formation of (H3/H4)_2_ tetramers on DNA. Thus, transition of H3/H4 from the Asf1-associated dimer to the DNA-associated tetramer is promoted by CAF-1-induced H3/H4 oligomerization.

## INTRODUCTION

The fundamental unit of chromatin is the nucleosome (1), which is composed of 147 base pairs (bp) of DNA wrapping 8 histone proteins, including 2 copies each of the core histones H2A, H2B, H3 and H4. Two H3/H4 dimers associate to form a tetrameric core, which is flanked by H2A/H2B dimers to form the nucleosome structure (2–4). Diverse groups of proteins, which include multi-subunit histone modification machines, ATP-dependent chromatin remodelers and histone chaperones, orchestrate the dynamic assembly and disassembly of nucleosomes that is critical for all DNA-dependent processes in the cell (reviewed in (5–7)). Histone chaperones bind directly to histones, directing histone transitions through distinct routes toward nucleosomal destinations (8,9). Anti-silencing function 1 (Asf1) is the central H3/H4 chaperone that accompanies H3/H4 to the nucleus (10). It also acts as a histone ‘sink’ by buffering the majority of non-nucleosomal H3/H4 (11,12). The route for nucleosome assembly after DNA synthesis and during repair involves the ‘handoff’ of H3/H4 from Asf1 to the downstream chaperone chromatin assembly factor 1 (CAF-1), which targets H3/H4 deposition to sites of DNA synthesis, where tetrasomes are formed (11,13,14).

From histone synthesis to deposition on DNA, the oligomerization state of H3/H4 is also regulated by histone chaperones. Asf1 binds tightly to a dimer of H3/H4 (*K*_D_ = 2.5 nM) (15–18), the predominant stoichiometric form of H3/H4 that exists in the eukaryotic cell (11). Ultimately, (H3/H4)_2_ tetramers are fashioned onto the DNA via CAF-1 and other proteins that target them to replication forks (5,19,20). The intrinsic preference of individual histone chaperones for tetrameric forms of H3/H4 is known for Nap1 and Vps75 (21), but not for CAF-1. As CAF-1 is the major H3/H4 chaperone involved in replication-dependent assembly, the nature of CAF-1–H3/H4 complexes may determine whether H3/H4 is deposited as (H3/H4)_2_ tetramers, or semi-conservatively as two distinct H3/H4 dimers onto newly synthesized DNA.

Three distinct regions of Asf1 contribute to protein–protein interactions. One face of the Asf1 N-terminal immunoglobulin-like core interacts with an H3/H4 dimer, whereas the opposite face interacts with a β-hairpin-like ‘B domain,’ present on CAF-1 (22) as well as Histone Regulatory Homolog A (HIRA) (23) and Rad53 (24). The C-terminal tail of Asf1 contributes interactions that promote Asf1 binding to Rad53 (24) and H3/H4 (25) (Dennehey *et al.*, manuscript submitted). However, the interactions of CAF-1 with Asf1 and H3/H4 are less well characterized. The three subunits that make up CAF-1 in budding yeast, Cac1 (p90), Cac2 (p60) and Cac3 (p48 or MSi1p), are moderately well conserved in eukaryotes. Whereas the Cac2 subunit harbors the B domain required to interact with Asf1 (22), the Drosophila and human homologs of Cac3, known as p55 (NURF), can independently interact with the N-terminus of H3 and the N-terminal α-helix of H4 (26–28). However, for H3/H4 to transition from Asf1 to CAF-1, the weak binding affinities that have been measured using truncated proteins and peptides necessitate other interactions between CAF-1 and Asf1 and H3/H4 that are currently unknown.

To gain insight into the interactions of the Asf1–H3/H4–CAF-1 axis, we developed and applied novel biophysical assays using the full-length recombinant proteins in the budding yeast system. A combination of electrophoretic mobility shift assays (EMSA) and fluorescence spectroscopy approaches provide quantitative evidence that the Asf1–CAF-1 interaction is indirectly enhanced by H3/H4 bound to Asf1, and directly by the C-terminal tail of Asf1. Furthermore, CAF-1 association with H3/H4 is mutually exclusive of Asf1, suggesting that CAF-1 functions independently of Asf1 after histone acquisition. Finally, EMSA, fluorescence spectroscopy, and chemical cross-linking analyses of the CAF-1–H3/H4 interaction reveals that a single CAF-1 complex binds to at least two H3/H4 dimers, ultimately promoting the formation of (H3/H4)_2_:DNA tetrasomes.

## MATERIALS AND METHODS

### Preparation of Proteins

The expression and inclusion body preparation of *Xenopus laevis* histones H3 and H4 with amino acid residue substitutions H3 C110A and H4 T71C, were as previously reported (18,29). Mutagenesis and plasmid construction methods are described in the Supplementary Methods. Position T71 in H4 was fluorophore labeled as previously described with either CPM (Invitrogen) or FM (Invitrogen) (18,29). To obtain dual-labeled CPM/FM-labeled H3/H4 populations, (H3/H4)_2_ tetramers were first individually labeled with either FM or CPM using standard procedures (29,30). Then, the H3/H4^FM^ and H3/H4^CPM ^complexes were mixed in equimolar concentrations and incubated for 60 min at 4°C. Purification and labeling of Asf1 and Asf1^1-169^ was performed as previously described (18,29).

CAF-1 was expressed and purified from baculovirus infected insect cells. Baculoviral transfer vectors were transfected along with the BD BaculoGold (BD Biosciences, Bright Linearized Baculovirus DNA (# 51-552846)) baculoviral backbone in Sf9 insect cells to produce viral stocks. Each viral stock was purified twice and protein expression was confirmed via western blotting. CAF-1 complex was produced by co-infecting Sf-9 cells at 37°C for 48 h using an MOI of 1 for each CAF-1 subunit. The Sf9 cell pellets were homogenized in a 1:1 ratio of pellet to buffer in 20 mM Tris (pH 8.0 at 4°C), 350 mM NaCl, 1 mM EDTA, 10 µg/ml of DNase I, complete protease tablet (Roche). Homogenate was clarified by centrifugation at 10 000× *g* for 30 min at 4°C. The supernatant of the clarified lysate was bound to a StrepTrap HP column (GE Healthcare) and washed extensively with 20 mM Tris (pH 8.0 at 4°C), 350 mM NaCl, 1 mM EDTA until the optical density (OD_280_) of the flow through reached baseline. CAF-1 complex was eluted from the column with 20 mM Tris (pH 8.0 at 4°C), 350 mM NaCl, 1 mM EDTA and 2.5 mM dsbiotin. CAF-1 was further purified by size-exclusion chromatography using a Superdex 200 column (GE Healthcare) in 10 mM Tris (pH 8.0 at 4°C), 350 mM NaCl, 1 mM EDTA and 1 mM DTT. Purified CAF-1 complex was concentrated with 10 000 MWCO centrifugal concentrators (Sartorius), aliquoted in small volumes, and flash frozen with liquid nitrogen.

Overexpression of pDEST-550-p60-His_6_ constructs was carried out in Rosetta pLysS cells (Novagen) grown in 1.6% bacto-tryptone, 1.0% yeast extract and 0.5% NaCl. Overnight cultures were used to inoculate 1 l cultures at 37°C. Cultures were induced at 0.8 OD_600_ with 0.4 mM IPTG and grown overnight (12–16 h) at 18°C. Harvested pellets were sonicated in 10 mM Tris (pH 8.0 at 4°C), 350 mM NaCl, 5 mM Imidazole, 15 mM BME, 10 µg/ml of DNase I and complete protease tablet (-)EDTA (Roche). The lysate was clarified by centrifugation at 10 000× *g* for 30 min at 4°C. The supernatant of the clarified lysate was bound to Ni-NTA resin (Qiagen) and washed extensively with 10 mM Tris (pH 8.0 at 4°C), 350 mM NaCl, 5 mM Imidazole, 15 mM BME until the OD_280_ of the flow through reached baseline. Cac2 protein was eluted from the column with 10 mM Tris (pH 8.0 at 4°C), 350 mM NaCl, 250 mM Imidazole, 15 mM BME using a stepwise gradient of 0–15% 1 column volume (CV), 15–70% 15 CV and 70–100% CV. Cac2 was further purified by size-exclusion chromatography using a Superdex 200 column (GE Healthcare) in 10 mM Tris (pH 8.0 at 4°C), 350 mM NaCl, 1 mM EDTA and 1 mM DTT. Purified Cac2 was concentrated with 10 000 MWCO centrifugal concentrators (Sartorius).

### Electrophoretic shift mobility assays

For resolution of protein–protein interactions, proteins were incubated on ice for 30 min in 10 mM Tris, 1 mM DTT and pH 8.0. The complexes were then separated on a pre-run 0.2× TBE 59:1 acrylamide:bis-acrylamide native gel for 90 min on ice. Gels were scanned on a Typhoon 9400 imager (GE Healthcare) for FM fluorescence (excitation at 488 nm, emission at 526 nm), followed by a scan for Alexa Fluor 532 fluorescence (ex. 532 nm, em. 555 nm). For western blots, antibodies against H3 (Abcam), Strep II (Novagen), FLAG (Abcam), His_6_ (Abgent) and Asf1 (Santa Cruz) were used. The unbound Asf1 species were identified according to the free Asf1 lane, whereas the bound species were slower migrating complexes. The integrated density value (IDV) of each band was quantified by ImageQuant (v. 5.2). To determine binding constants, fraction bound was defined according to Equation (1) and the *K*_D_ calculated according to Equation (2) by GraphPad Prism (v. 5.0d).
(1)
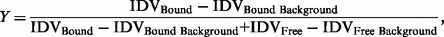

(2)
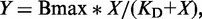

where Bmax is maximum binding, and *X* is the concentration of Cac2 or CAF-1.

The protocol to analyse histone deposition onto DNA was followed as previously described (18,29). Briefly, histones H3/H4 and chaperone(s) were allowed to interact in 10 mM Tris, 150 mM NaCl, 0.5 mM TCEP, pH 8.0 on ice for 20 min, followed by interaction with DNA on ice for 20 min. The complexes were separated on a pre-run 0.2× TBE gel for 150 min on ice. Gels were scanned for FM fluorescence, then stained with 1:10 000 diluted Sybr Green I (Invitrogen) and re-scanned using the same parameters. To quantify the percentage of disomes formed, the IDV of the disome and tetrasome bands computed by ImageQuant were used to calculate Equation (3).
(3)
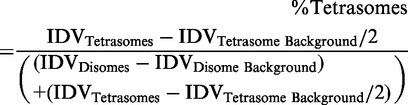



### Fluorescence spectroscopy

For fluorescence anisotropy, 10 nM of Alexa Fluor 532-labeled Asf1 or 100 nM labeled Asf1^1-169^ was allowed to equilibrate in 20 mM HEPES, 150 mM NaCl, pH 7.4 in a FluoroLog-3 (Horiba) spectrofluorometer equipped with a thermostat set at 20°C. Cac2 or CAF-1 was then titrated; at each point after equilibration, Alexa Fluor 532 was excited at 528 nm (slit width: 6 nm) by vertically and horizontally polarized light, while the intensity (slit width: 13 nm) of polarized light (emission 548 nm) was also monitored in the vertical and horizontal planes. The anisotropy was calculated according to Equation (4) and the *K*_D_ was calculated by Equation (2).
(4)
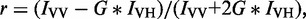

where *G* is the grating factor (*G* = *I*_HV_/*I*_HH_) used to correct for fluorometer sensitivity to polarization bias.

For FRET (fluorescence resonance energy transfer) experiments, 2 nM of H3/H4 mix labeled with CPM or FM were equilibrated in 10 mM Tris, 150 mM KCl, 2 mM MgCl_2_, 1% glycerol, 0.5 mM TCEP, 0.05% BRIJ-35 at 20°C. To evaluate FRET, CPM was excited at 385 nm, and the emission spectrum recorded from 400–600 nm. In parallel, FM was excited at 491 nm, and the emission recorded from 500–600 nm. The FRET Ratio was calculated from the CPM excited spectrum according to Equation (5).
(5)
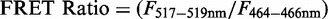



Because we observed a quench of the entire spectrum upon addition of an H3/H4 binding partner, we used the FRET ratio for estimation of the H3/H4–CAF-1 *K*_D._ FRET Ratios were normalized to the Fmax to obtain the fraction of bound H3/H4. A ligand-depleted binding equation was used to fit the fluorescence data binding models:
(6)
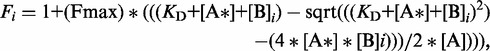

where *i* indicates the varying concentrations of unlabeled protein B that were titrated into the labeled protein A*.

### Chemical cross-linking

DSS (Thermo Pierce) was prepared at 10 mM by dissolution in DMSO. 1 µM CAF-1 and 2 µM H3/H4 dimers were allowed to incubate with 500 µM DSS or DMSO for 30 min at room temperature in 20 mM HEPES, 150 mM NaCl, pH 7.4. The cross-linking reaction was quenched by addition of 50 mM Tris pH 7.4, and incubated for an addition 15 min. The reactions were electrophoresed on a 4–15% Tris–HCl gel (Criterion), stained with Coomassie Blue or immunoblotted for the relevant proteins.

### Size-exclusion chromatography

A Superdex 200 16/60 (GE Healthcare) size-exclusion column was used for chromatography. 5.3 µM of CAF-1 with or without 10.6 µM H3/H4 dimers were incubated on ice for 30 min in 50 mM KH_2_PO_4_, 100 mM KCl, 5 mM DTT, pH 7.2. The proteins were then run through a Superdex 200 size-exclusion column in the same buffer at a flow rate of 0.5 ml/min. The elution was monitored by absorbance at 280 nm.

## RESULTS

### The CAF-1–Asf1 complex is stabilized through the C-terminus of Asf1

**Figure 1.**
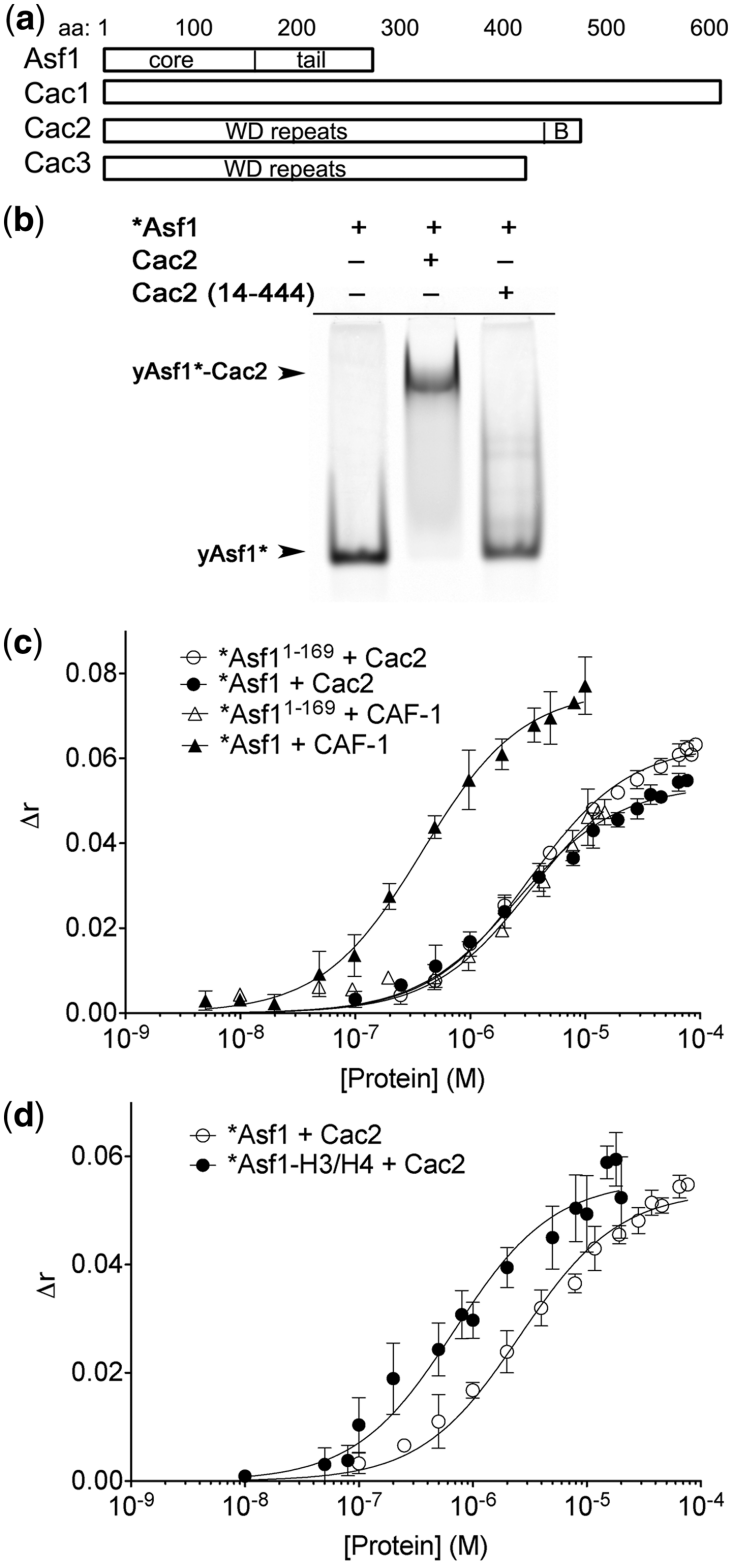
The C-terminal tail of Asf1 participates in CAF-1 interactions. (**a**) Schematic diagram of Asf1 and CAF-1 domains relevant to binding studies. (**b**) EMSA showing 0.5 µM of Asf1 labeled with Alexa Fluor 532 (*Asf1), *Asf1 bound to 20 µM Cac2 lacking the B domain, and *Asf1 bound to 20 µM full-length Cac2. (**c**) Fluorescence anisotropy of 10 nM *Asf1 or 100 nM of *Asf1 with a shortened C-terminal tail (*Asf1^1-169^), bound to increasing concentrations of Cac2 or CAF-1. (**d**) Fluorescence anisotropy of 10 nM *Asf1 pre-bound to 20 nM H3/H4, then titrated with Cac2. The data were fitted using Equation (2) and show the mean and standard deviation from at least three independent experiments.

Asf1 and CAF-1 interact directly through the B domain of CAF-1 subunit Cac2, an interaction thought to facilitate H3/H4 transfer between the chaperones. The *K*_D_ of this interaction was previously determined to be ∼1.5 µM, but because this binding affinity was measured with only a B domain peptide (22) (Figure 1a), we studied the interaction with the full-length proteins and the intact CAF-1 complex. The binding of fluorophore labeled *Asf1 (Alexa Fluor 532 located at a cysteine that was engineered at the N-terminus at the ‘−1’ position of the of Asf1 sequence, Supplementary Figure S1a and b) to 20 µM of either full-length Cac2 or Cac2 lacking the B domain was monitored using EMSA. Free *Asf1 migrates with a higher relative mobility than the *Asf1–Cac2 complex under non-denaturing conditions, resolving bound and unbound species (Figure 1b). Using this system, full-length Cac2, but not Cac2 lacking the B domain, was able to retard the mobility of *Asf1. To measure the binding constant between Cac2 and *Asf1, we titrated Cac2 into *Asf1 (Supplementary Figure S2a) and determined the *K*_D_ to be 2.0 µM (Supplementary Figure S2c and Table 1), in agreement with a previous study using a B domain peptide (22). As the EMSA method is susceptible to dissociation of the components during electrophoresis, we also used fluorescence anisotropy to measure the binding affinity (Figure 1c). The anisotropy of *Asf1 increased with Cac2 titration, consistent with formation of the Asf1–Cac2 complex. The *K*_D_ determined using this method was 2.6 µM (Table 1). These results demonstrate that intact Asf1 and Cac2 form a stable complex with modest low micromolar binding affinity, but only when the Cac2 B domain is present (22,23).

**Table 1.** Binding affinities of Asf1 for Cac2 and CAF-1 and CAF-1 for H3/H4

Protein or complex	Binding partner	*K*_D_ from EMSA (M)	*K*_D_ from fluorescence anisotropy (M)	*K*_D_ from FRET (M)
*Asf1	Cac2	2.0 (±1.0) × 10^−6^	2.6 (±0.6) × 10^−6^	
*Asf1	Cac2^14-444^	n.o.	n.a.	
*Asf1^1-169^	Cac2	n.o.	3.3 (±0.6) × 10^−6^	
*Asf1	CAF-1	2.8 (±0.4) × 10^−7^	3.8 (±0.9) × 10^−7^	
*Asf1^1-169^	CAF-1	n.o.	3.4 (±0.5) × 10^−6^	
*Asf1–H3/H4	Cac2		6.8 (±0.9) × 10^−7^	
H3/H4*	CAF-1	1.9 (±1.1) × 10^−8^	n.a.	5.2 (±1.5) × 10^−9^
H3^110E^/H4*	CAF-1	n.a.	n.a.	3.5 (±0.4) × 10^−9^

*Labeled protein; n.o., not observed; n.a., not attempted.

In order to determine whether the association of Asf1 with CAF-1 is mediated solely through the Cac2 subunit, we determined the binding affinity of Asf1 for the intact CAF-1 complex. CAF-1 stably interacts with *Asf1 in both the EMSA (Supplementary Figure S2b) and fluorescence anisotropy assays, allowing for measurement of binding affinities. The *K*_D_ was 0.28 µM (Supplementary Figure S2c and Table 1) and 0.38 µM (Figure 1c and Table 1) measured by EMSA and fluorescence anisotropy, respectively. Surprisingly, the affinity of Asf1 for the entire CAF-1 complex is ∼10-fold greater than for the Cac2 subunit alone, which is indicative of novel interactions between these two histone chaperones.

Recent studies have shown that the Asf1 C-terminal tail contributes to interactions with binding partners, notably with Rad53 and H3/H4 (24,25). To determine whether this domain also promotes the association of Asf1 with CAF-1, we tested the binding of a C-terminally truncated Asf1 (Asf1^1-169^) that lacks the majority of the C-terminal tail. Because the fluorophore labeled *Asf1^1-169^–Cac2 and *Asf1^1-169^–CAF-1 complexes are poorly resolved in the EMSA (data not shown), fluorescence anisotropy was used to derive the binding constants (Figure 1c). Full-length Asf1 and Asf1 lacking the C-terminal tail bind with similar affinity to Cac2 (*K*_D_ ∼3 µM) (Table 1). The 10-fold increase in binding affinity occurred only upon titration of the tri-subunit CAF-1 complex with full-length Asf1 (Figure 1c), which suggests a direct interaction between the Asf1 tail and CAF-1, exclusive of the Cac2 subunit.

### The Cac2–Asf1 complex is stabilized through Asf1-bound H3/H4

H3/H4 and Cac2 do not bind directly *in vitro* (31), and are positioned on opposite surfaces of the Asf1 Ig-like core, yet several lines of evidence suggest that H3/H4 may render Asf1 more amenable for binding Cac2. First, glycerol gradient sedimentation experiments showed that the presence of H3/H4 markedly enhances the formation of a stable Asf1–CAF-1 complex, although H3/H4 was not confirmed to be included in this complex (32). Intriguingly, the crystal structure of Asf1–H3/H4 reveals H3/H4-induced structural changes in Asf1 in the vicinity of residues 35–77 (16). Accordingly, alanine substitutions of D37 or L60/V62 abolish binding between the globular core of Asf1 and Cac2 B domain peptides (22). To examine the influence of H3/H4 on the interaction of Asf1 with Cac2, we compared the affinity of Cac2 for *Asf1–H3/H4 complexes to that of *Asf1 alone (Figure 1d and Table 1). Our results show that Asf1 in complex with H3/H4 binds ∼3-fold tighter to Cac2 (0.7 µM) than Asf1 alone. Because the Cac2 subunit does not bind H3/H4 directly, the stronger interaction is possibly caused by conformational changes on Asf1 induced by H3/H4 (Supplementary Figure S6). Together, the binding data reveal that the Asf1–CAF-1 complex is stabilized through two previously uncharacterized mechanisms.

### Asf1 dissociates from CAF-1–H3/H4 complexes

**Figure 2.**
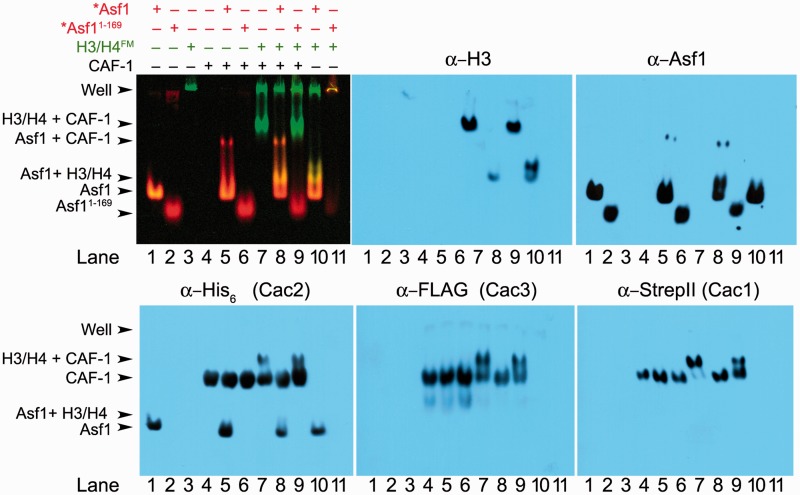
Asf1 and CAF-1 form mutually exclusive complexes with H3/H4. A representative EMSA and western blots showing complexes of Alexa Fluor 532-labeled Asf1 (*Asf1), fluorescein-labeled H3/H4 (H3/H4^FM^), and unlabeled CAF-1 resolved using native gel electrophoresis, and the appropriate fluorophores detected on a Typhoon 9400 imager. Detection of *Asf1 was set to display as red, whereas H3/H4 ^FM^ was set to display as green. To validate the identity of proteins in each complex, immunoblots using the indicated antibodies were performed. α-His_6_ was used to probe for Cac2, α-FLAG for Cac3, α-Strep II for Cac1, α-Asf1 for Asf1 and α-H3 for histone H3.

That the C-terminal tail of Asf1 exhibits interactions with both CAF-1 and H3/H4 raises the possibility that this domain may stabilize the formation of a larger complex containing all three complexes together, thereby facilitating H3/H4 transfer from Asf1 to CAF-1. Although Rad53 also interacts with the Asf1 C-terminus, a recent biochemical study reported that Rad53 and H3/H4 binding to Asf1 are competitive (24). Thus, it is also possible that the interaction of Asf1 with histones is competitive with CAF-1. To examine the complexes that are formed with Asf1, H3/H4 and CAF-1, we developed an EMSA approach capable of resolving these distinct complexes. We allowed equimolar (250 nM) concentrations of *Asf1 or *Asf1^1-169^, H3/H4 with fluorescein (FM) labeled on Cys 71 of H4 (H3/H4^FM^), and unlabeled CAF-1 to interact with each other, and then separated the complexes by native gel electrophoresis. The constituents of the bands were subsequently analysed using fluorescence imaging and immunoblotting for each of the relevant proteins (Figure 2). Each recombinant CAF-1 subunit was immunoblotted for the respective C-terminal tags: Cac1 was tagged with Strep II, Cac2 with His_6_ and Cac3 with FLAG. In Figure 2, *Asf1 (lane 1), *Asf1^1-169^ (lane 2), CAF-1 (lane 4) and CAF-1–H3/H4^FM^ complexes (lane 7) can be observed. However, *Asf1 was not seen to associate with CAF-1 in the absence (lanes 5 and 6) or presence (lanes 8 and 9) of H3/H4^FM^, which is consistent with the moderate binding affinity we observed earlier (Figure 1).

The EMSA experiments reveal competitive interactions between Asf1 and CAF-1 for H3/H4. When *Asf1^1-169^ was incubated with H3/H4^FM^ and CAF-1, CAF-1 predominantly associates with H3/H4^FM^ (Figure 2 lane 9). The fluorescence of H4 together with the immunoblot for H3 demonstrates that an intact H3/H4 complex binds to CAF-1. This complex is not observed, however, when full-length *Asf1 is present, (Figure 2 lane 8), suggesting that the binding affinity of CAF-1 for H3/H4 ^FM^ is weaker than Asf1 for H3/H4^FM^, yet greater than *Asf1^1-169 ^for H3/H4^FM^. Importantly, even with the stabilizing effects of the C-terminal tail on H3/H4^FM^ and CAF-1, all three components, *Asf1, H3/H4^FM^ and CAF-1, do not form a stable complex together. Instead, we only observed H3/H4^FM^ in association with one chaperone at a time, although transient interactions cannot be detected in our system. Therefore, H3/H4 association with CAF-1 is mutually exclusive of the Asf1–H3/H4 interaction.

### CAF-1 exists primarily in the monomeric form in the presence and absence of H3/H4

**Figure 3.**
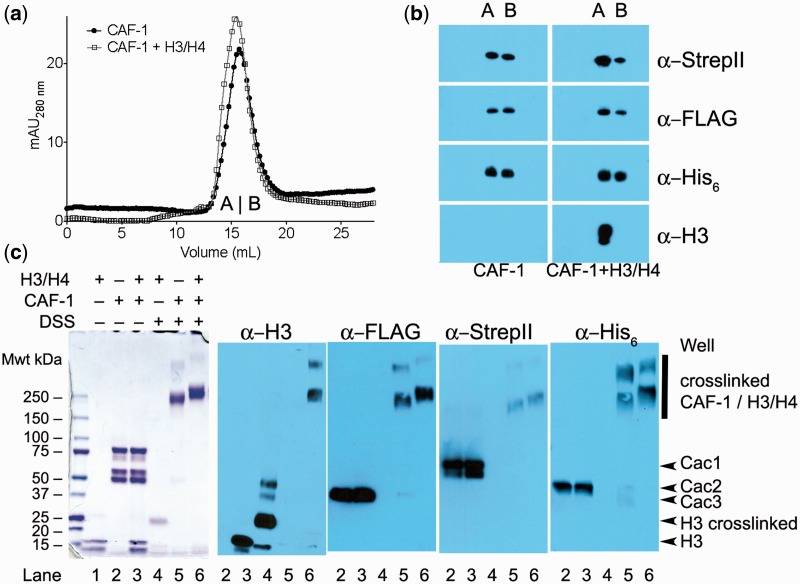
CAF-1 interacts primarily in the monomeric form with H3/H4. (**a**) Size-exclusion chromatography. 5.3 µM CAF-1 alone or in complex with 10.6 µM H3/H4 was eluted from a 30 ml Superdex 200 column. 2 ml fractions were collected throughout the run. Fractions A and B designate elution volumes 14–16 and 16–18 ml, respectively. (**b**) Western blot identification of the species found in fractions A and B from the SEC in panel (a) (CAF-1 left-hand side, CAF-1 with H3/H4 right-hand side). (**c**) Cross-linking CAF-1 and H3/H4 with DSS. 4–12% gradient SDS PAGE gels resolve samples of 1 µM CAF-1, 2 µM H3/H4 and 500 µM of DSS incubated together as indicated. The gels were then stained with Coomassie Blue or western blotted for the relevant proteins using the same antibodies as in Figure 2.

Biochemical studies suggest that *X. laevis* and *Homo sapiens* CAF-1, or its subunit Cac1, may exist in a monomer/dimer equilibrium, with the human monomer form displaying preferential binding to PCNA (20,33). Therefore, the oligomeric state of CAF-1 may influence binding to histones H3/H4. To look for such changes in molecular weight, we used analytical size-exclusion chromatography with CAF-1 with and without H3/H4. CAF-1 at a concentration of 5.3 µM elutes as a single complex, without any indication of higher stoichiometric forms (Figure 3a and b). Importantly, when CAF-1 was incubated with a 2-fold concentration of H3/H4, the elution profile shifts slightly to higher molecular size, but remains suggestive of a monomeric form of CAF-1 associated with H3/H4. Again, there was no appearance of peaks eluting at smaller elution volumes. However, because molecular weight determination relies on comparison to globular standard proteins, this technique does not give reliable molecular weight estimations for asymmetric proteins.

Thus, to further determine the stoichiometric state of the CAF-1:H3/H4 association, a chemical cross-linking approach was employed. 1 µM CAF-1 and 2 µM H3/H4 were incubated with 500 µM disuccinimidyl suberate (DSS), which chemically links primary amines within 11.4 Å proximity. The cross-linked complexes were resolved by SDS-PAGE, followed by immunoblotting for each of the CAF-1 subunits and histone H3 (Figure 3c). Distinct CAF-1 and CAF-1:H3/H4 cross-linked complexes migrate with mobilities around the 250 kDa molecular weight standard. CAF-1 with epitope tags has a predicted mass of 171.6 kDa, and a predicted complex of one CAF-1 and a (H3/H4)_2_ tetramer is 225 kDa. There are minor, slower migrating species containing both CAF-1 and H3/H4, which are most likely multimers of the complexes representing the dimeric form of CAF-1. However, the major mode of binding with saturating binding concentrations in this assay involves a monomer of one CAF-1 complex. Together, these data indicate that baculovirus-expressed *Saccharomyces cerevisiae* CAF-1 exists as a monomeric complex in the absence and presence of bound H3/H4.

### CAF-1 associates with multimers of H3/H4

**Figure 4.**
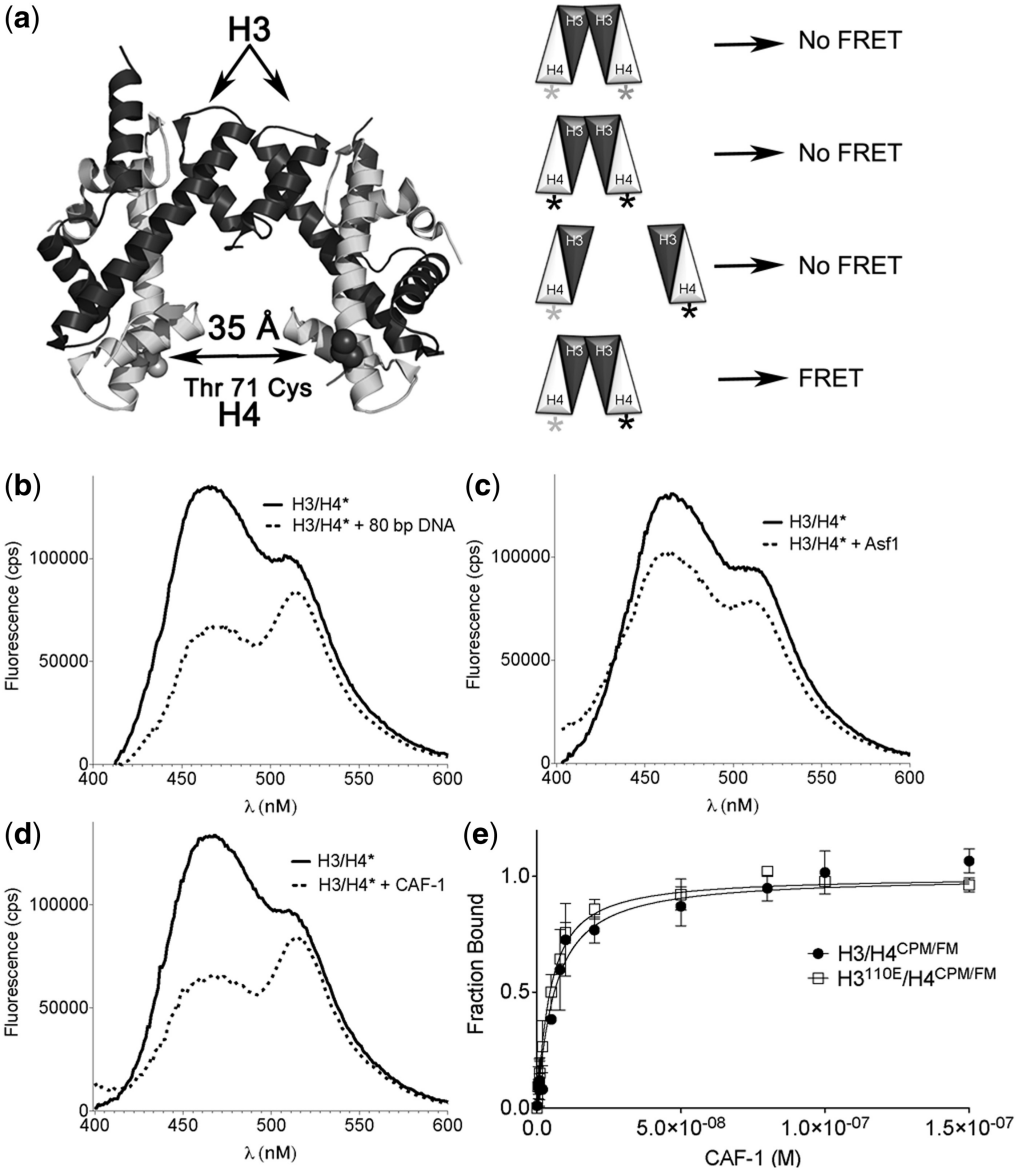
CAF-1 induces the formation of H3/H4 oligomers. (**a**) A FRET approach to report H3/H4 tetramerization. Positioning of fluorophores FM and CPM used for FRET experiments on the cysteine 71 residue of H4. H3/H4 are presented here as tetramers. A mixed labeled population of H3/H4, composed of H3/H4 with FM at Cys^71^ on H4 and CPM at Cys^71^ on H4, reports FRET when CPM is excited at 385 nm and when the CPM–FM donor–acceptor pair is within close range. (**b**) 10 nM H3/H4^CPM/FM^ under dimerizing conditions (20°C, Buffer = 10 mM Tris pH 7.5, 150 mM KCl, 2 mM MgCl, 1% glycerol, 0.5 mM TCEP, 0.05% BRIJ-35) display FRET in the presence of 100 nM 80 bp 601 DNA. (**c**) 100 nM of unlabeled Asf1 bound to 10 nM H3/H4^CPM/FM^ does not result in FRET. (**d**) 100 nM CAF-1 bound to 10 nM H3/H4^CPM/FM^ results in CPM–FM FRET to the same degree as H3/H4 tetramerization on 601 DNA. (**e**) Using the FRET system, the binding affinity of CAF-1 to H3/H4^CPM/FM^ or H3^110E^/H4^CPM/FM^ was determined by monitoring the FRET ratio (F_517-519 nm_/F_464-466 nm_) with each CAF-1 titration. These data were fitted using Equation (6) and show the mean and standard deviation from at least three independent experiments.

To gain quantitative insights into the nature of the CAF-1–H3/H4 interaction, EMSA and FRETexperiments were developed and used to measure binding affinity. The FRET approach examines a mixed labeled population of H3/H4, in which one half of the H4 protein was labeled with the FRET acceptor FM at Cys 71, whereas the other half was labeled with the FRET donor 7-Diethylamino-3-(4′-Maleimidylphenyl)-4-Methylcoumarin (CPM) at the same site (Figure 4a). This approach predicts FRET to be observed between CPM and FM upon CPM excitation, but only when the FRET donor and acceptor are close together.

Under conditions of high ionic strength, H3/H4 is well known to adopt a tetrameric configuration (18,34,35). Using these conditions in our FRET system, a high FRET ratio (ratio of the FM to CPM fluorescence emission upon CPM excitation) is observed (Supplementary Figure S3a). Addition of guanidine HCl decreases this ratio and is consistent with the denaturation of the H3/H4 tetramer (Supplementary Figure S3b), similar to previous observations of the stability of H3/H4 in the presence of chemical denaturants (36). Interestingly, even at concentrations of 2M NaCl, addition of Asf1 decreased the FRET ratio (Supplementary Figure S3a), indicating that Asf1 can disrupt the H3/H4 tetramer. Analysis of a titration series comparing the change in FRET signal to the change in Asf1 tryptophan fluorescence gave nearly identical binding curves with an estimated *K*_D_ of 50 nM (Supplementary Figure S3c). As a control, Asf1 substituted at valine 94 with arginine (Asf1V94R), which is known to disrupt the Asf1–H3/H4 interaction (37), showed considerably weaker binding in this assay.

Under conditions of low salt concentrations that favor H3/H4 in dimer form (18), FRET was not observed, allowing possible detection of H3/H4 oligomerization. To test this idea, mixed labeled histones were allowed to equilibrate and then associate with 10-fold molar excess (100 nM) of 80 bp 601 DNA (18,29,38). This histone:DNA interaction, which highly favors (H3/H4)_2_:DNA tetrasome formation, causes an increase in the FRET ratio from the basal 0.7 ratio to 1.3, generating a FRET effect (Figure 4a and Supplementary Figure S4a–c). In contrast, an H3/H4^CPM/FM^ interaction with 10-fold molar excess (100 nM) full-length Asf1, which binds to H3/H4 dimers, does not change the FRET ratio or FRET effect under identical conditions (Figure 4b and Supplementary Figure S4a–c). Therefore, this fluorophore configuration reveals changes in the proximity of H3/H4 dimers and therefore changes in H3/H4 oligomerization.

In order to determine whether CAF-1 associates with one H3/H4 dimer or an oligomeric form of H3/H4, we analysed H3/H4^CPM/FM^ FRET upon CAF-1 addition. Figure 4c shows that the fluorescence emission of the histones bound to 100 nM CAF-1 is almost identical to the shape of the fluorescence emission spectrum of H3/H4 bound to DNA. Importantly, the FRET ratio and the FRET effect for both the CAF-1–H3/H4 and H3/H4–DNA complexes are nearly the same. Although the EMSA (Supplementary Figure S5) gave a dissociation constant of 19 nM, using the FRET ratio to monitor titrations of CAF-1 to H3/H4^CPM/FM^ (Supplementary Figure S4d) reported a *K*_D_ of 5.0 nM (Table 1). This *K*_D_ value is indeed between those of Asf1–H3/H4 and Asf1^1-169^–H3/H4, which is consistent with predictions based on Figure 2 and known Asf1–H3/H4 affinities (18,29). As the FRET signal of H3/H4^CPM/FM^ associated with DNA and CAF-1 was similar, the expected inter-fluorophore distances would also be expected to be similar in the two complexes, which suggests that H3/H4 dimers adopt similar configurations when bound to DNA and to CAF-1. In order to investigate whether the arrangement of H3/H4 bound to CAF-1 was identical to the *bona fide* tetramer observed in nucleosomes, an H3/H4 tetramerization defective mutant was analysed. H3 was substituted at position 110 to glutamate, an acidic residue known to disrupt the hydrophobic tetramerization interface (1,39). The binding affinity of CAF-1 to H3^110E^/H4^CPM/FM^ CAF-1 was 3.5 nM, similar to WT H3/H4 (Figure 4d). Therefore, the oligomerization of H3/H4 on CAF-1 can tolerate the H3^110E ^substitution, suggesting that the H3/H4 oligomer bound by CAF-1 may not be a canonical (H3/H4)_2_ tetramer.

### CAF-1 deposits (H3/H4)_2_ tetramers on DNA

**Figure 5.**
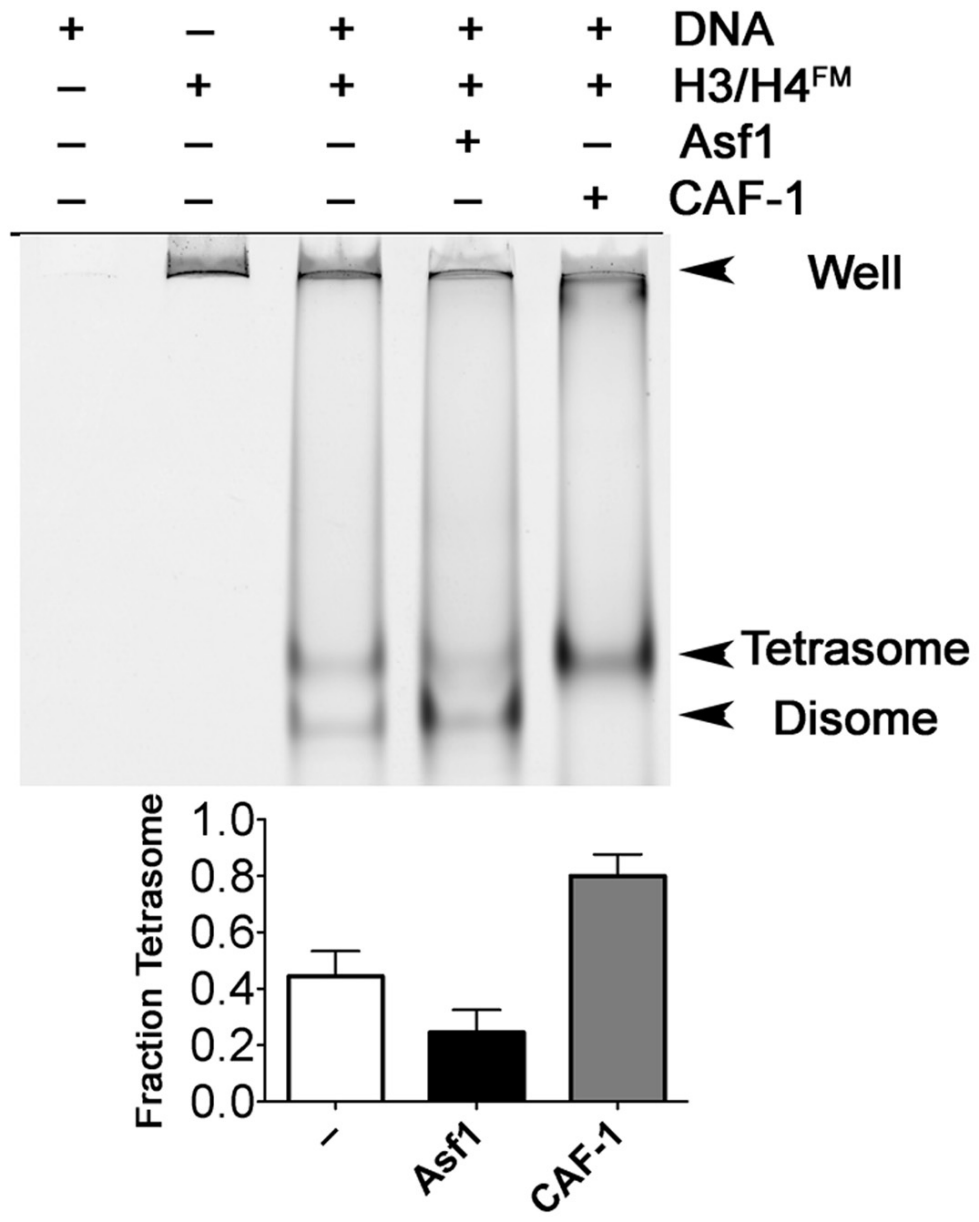
CAF-1 enhances the formation of tetrasomes. An EMSA system was used to separate disome and tetrasome species formed on 80 bp 601 DNA. 0.2 µM of H3/H4^FM^ was incubated with 1.6 µM Asf1 or CAF-1, followed by addition of 0.4 µM DNA. The gels were scanned using the Typhoon 9400 imager and fraction of tetrasome values were obtained using Equatioin (3). The mean and standard deviation from at least three independent experiments are shown.

Even if CAF-1 does not bind to canonical (H3/H4)_2_ tetramers, our FRET experiments (Figure 4) indicate that it primes H3/H4 in an intermediate configuration closer to the final tetramer form than Asf1-bound dimers. Therefore, CAF-1 would be predicted to deposit H3/H4 as tetramers rather than dimers onto DNA. To test this prediction, we utilized an EMSA approach recently described (18,29), in which H3/H4^FM^ associated with histone chaperones are allowed to be deposited onto DNA, and the resulting complexes resolved by native gel electrophoresis (Figure 5). Detection of H3/H4 and DNA by FM fluorescence and Sybr Green I staining, respectively, distinguish between dimers of H3/H4 formed with DNA (disomes), and tetramers formed with DNA (tetrasomes). This method was previously used to show that, at near micromolar concentrations, H3/H4:DNA complexes primarily form tetrasomes *in vitro*, but that Asf1 activity dramatically induces formation of disomes (18). Using this approach, we altered the molar H3/H4:DNA ratio such that 0.2 µM H3/H4 association with 0.4 µM DNA yields 44% tetrasomes. At this percentage, alteration of the disome/tetrasome equilibrium mediated by a histone chaperone would be readily evident. As expected, Asf1 reduces the tetrasome percentage to 24%, whereas CAF-1 increases it to 80% (Figure 5). This suggests that CAF-1 favors the formation of (H3/H4)_2_:DNA tetrasomes.

## DISCUSSION

**Figure 6.**
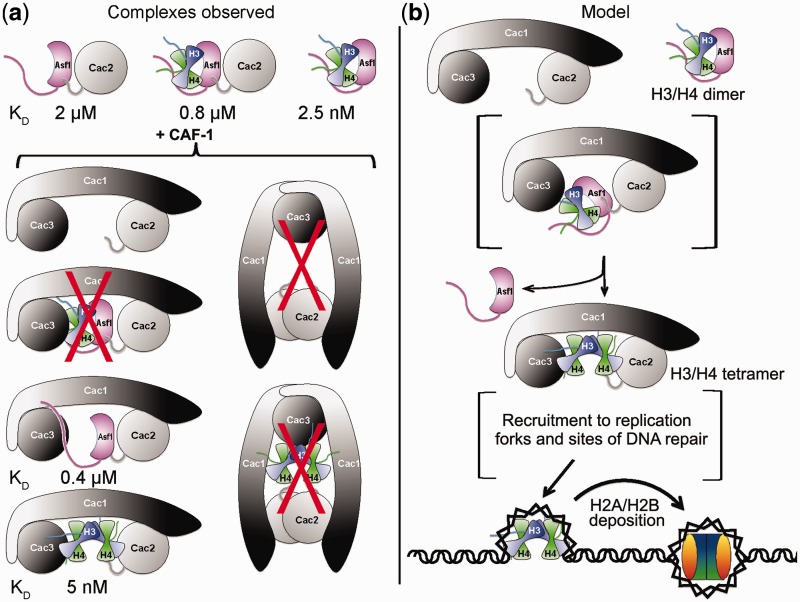
Revised model of H3/H4 transitions among Asf1 and CAF-1 histone chaperones. (**a**) Schematic diagram showing the complexes observed in this study and others. Complexes that are not observed are marked with a red X. *K*_D_ values are shown for those measured in this system. (**b**) Model of H3/H4 associations with Asf1 and CAF-1. The histones are in dimer form when bound to Asf1. Even though Asf1 and Asf1–H3/H4 can associate with Cac2, they were not found in a stable complex with intact CAF-1. Instead CAF-1 binds to multimers of H3/H4 and deposits them as tetramers on DNA to form tetrasomes.

The paths that histones follow and the states they adopt from synthesis to deposition on DNA are important for their correct nucleosome dynamics, but a detailed understanding of the physical basis of this process has lagged *in vivo* studies. We and others showed that the H3/H4 chaperone Asf1 binds to the dimer form of H3/H4 (11,15–17) with an affinity of ∼2 nM (18). However, the strengths and specificity of many of the interactions involved in the encounter of either Asf1, the Asf1–H3/H4 complex, or H3/H4 with the replication-dependent histone chaperone CAF-1 remained unknown. The biophysical analyses reported here for Asf1, H3/H4, CAF-1 and Cac2 provide unique insights into the states that these chaperones and histones can adopt (Figure 6a) and demonstrate that CAF-1 has intrinsic H3/H4 tetamerization activity, thereby supporting a revised model for nucleosome assembly (Figure 6b).

### Novel mechanisms stabilize the Asf1–CAF-1 complex

We found evidence for novel interactions of Asf1 with CAF-1 that occur through the C-terminal tail of Asf1 with CAF-1 subunits other than Cac2. Previous studies showed that the N-terminal domains of human Asf1a and *Schizosaccharomyces pombe* Asf1 bind to the Cac2 protein via the B domain with micromolar binding affinity (22,23). Our biophysical analyses (Figure 1b and c, Table 1 and Supplementary Figure S2a and c) are consistent with these observations, and together show how conserved the interactions of Asf1 and Cac2 are among species. However, beyond the moderate strength (micromolar) interactions of Asf1 with Cac2, these newly discovered interactions between the Asf1 C-terminal tail and CAF-1 further stabilize the complex by 10-fold, driving the affinity into the nanomolar range (Figure 1c and Supplementary Figure S2b and c). The observation that the C-terminal tail of Asf1 has a modulatory role in the association of Asf1 with CAF-1 might also extend to the different Asf1 orthologs, such as the human Asf1a and Asf1b isoforms that have differential selectivity for recognition of CAF-1 and HIRA (13).

We found that Asf1 pre-bound to H3/H4 associates with Cac2 more tightly than Asf1 alone. The only known contacts of Asf1, with Cac2 occur through the Asf1-B domain binding site, which is on the opposite face of Asf1 from the histone binding site. Thus, the simplest explanation is that H3/H4 induces a structural change that permits tighter Asf1–Cac2 binding. Indeed, alignment of this region with free Asf1, B domain-Asf1 and Asf1–H3/H4 structures show a distinct difference between the H3/H4-bound complexes and all of the others in the B-domain binding site, even though the crystal packing environments and species are all different (Supplementary Figure S6) (16,17,22,23,40,41). Therefore, we suggest that Asf1 loaded with H3/H4 interacts more strongly with Cac2 than Asf1 alone does While a H3/H4–Asf1–Cac2 complex is likely a transient one *in vivo*, we suggest that it may reflect an intermediate step in the process of transfer of H3/H4 between histone chaperones (Figure 6).

### CAF-1 binds to multiple H3/H4 dimers with nanomolar affinity

Asf1 does not remain as a stable component in the CAF-1–H3/H4 complex. Although Asf1 has been identified in complexes with CAF-1 *in vivo* (42), it was not known whether CAF-1–Asf1–H3/H4 can form a thermodynamically stable complex , and our analysis failed to detect the six proteins together in the same complex. Our results are also consistent with recent biochemical data, in which Asf1 could not be detected in CAF-1 immunoprecipitates from human colon carcinoma cells (43), suggesting that Asf1–H3/H4 and CAF-1–H3/H4 species are distinct complexes. CAF-1 activity with H3/H4, then, is independent of Asf1, requiring only the transfer of histones from Asf1.

CAF-1 complexes with and without bound H3/H4 exist primarily in monomeric form. Previous studies observed formation of dimers of the human homolog of the yeast Cac1 subunit (33), but the oligomerization state of CAF-1 in the absence or presence of H3/H4 was not known. A single species of CAF-1, most consistent with a monomer, was observed under all of the conditions tested, including SEC and covalent cross-linking (Figures 2–4). We conclude that CAF-1 does not undergo dimerization upon binding of H3/H4 (Figure 6a), and that the monomer form is competent for this interaction.

Unexpectedly, H3/H4 binds more tightly to Asf1 than to CAF-1. We suggested that histone chaperones serve to guide histones down thermodynamically favorable pathways toward their final and presumably most thermodynamically stable destination in the cell, the nucleosome (8). Therefore, we were surprised to find that CAF-1 binds to H3/H4 with a 2-fold weaker affinity than does Asf1 (Figures 2 and 4, and Table 1). To execute H3/H4 ‘handoff,’ the binding affinities between Asf1–H3/H4 or CAF-1–H3/H4 are most likely modulated by post-translational modifications. Accordingly, numerous studies support the role of histone modifications in facilitating histone transfer among chaperones ((44–46) reviewed in (13)). In fact, biochemical analyses suggest that the Cac1 subunit of CAF-1 preferentially interacts with H3/H4 that has been acetylated on lysine 56 of H3 (31).

Prior to the surprising finding that Asf1 binds to a dimer of H3/H4 in the cell, the assumption was that H3/H4 existed predominantly as tetramers. Our previous studies (18) as well as results presented here (Figure 4 and Supplementary Figure S3) show that under conditions of physiological ionic strength and sub-micromolar concentrations, H3/H4 exists in a dynamic equilibrium between dimers and tetramers. So the question then became: How do H3/H4 dimers tetramerize so that they can be deposited onto DNA as a correctly formed tetrasome? The results of mixed species FRET experiments and tetrasome formation assays point to the formation of the CAF-1–H3/H4 complex as the pivotal step in this process. The nearly identical FRET signal, observed for H3/H4 bound to DNA or CAF-1, argues strongly that H3/H4 adopts a similar configuration in each complex, but whether this is a true H3/H4 tetramer is not known, as the precise structure of H3/H4 in the tetrasome or in CAF-1 is not known.

The similar binding affinity of CAF-1 for H3^110E^/H4, a mutant that weakens the (H3/H4)_2_ tetramerization interface (39), argues that H3/H4 is not arranged as a canonical tetramer on CAF-1. Unfortunately, the direct fluorophore quenching we observed precludes the determination of inter-fluorophore distances, which could otherwise have been compared to distances within the nucleosome. Certainly, the interactions within CAF-1 that promote oligomerization are unlikely to be symmetric, as the Cac3 subunit of CAF-1 binds to a dimer of H3/H4 (26,28), and the Cac1 subunit likely interacts with the N-terminal helix of H3 (31). Such a complex represents a previously unrecognized asymmetric histone binding mode among histone chaperones, since all other chaperones that bind to multimers of H3/H4, including Nap1, Vps75 and Rtt106, dimerize themselves (21,45,47). The finding that H3/H4 oligomerizes in a manner that closely resembles its final form in a tetrasome, nevertheless, supports the notion that CAF-1 uniquely positions H3/H4 in an intermediate form primed for tetramerization on DNA.

CAF-1 functions at a pivotal position in the assembly of newly synthesized H3/H4 onto DNA and the chaperoning of parental histone H3/H4 from replicating DNA, operating in such a way as to prevent new and parental forms of H3/H4 from mixing within nucleosomes (14,19,48). This occurs despite the dimeric form of H3/H4 that is delivered to CAF-1 by Asf1. The simplest mechanism by which this can be accomplished would be for CAF-1 to have an active role in tetramerizing newly synthesized H3/H4 received from Asf1, and to have the capacity to bind to parental H3/H4 tetramers for reassembly after replication fork passage. Indeed, this is the mechanism uncovered here. Moreover, these findings provide unique insights into the states that these chaperones and histones can adopt, and point to additional mechanisms that can fine tune the process of nucleosome assembly.

## SUPPLEMENTARY DATA

Supplementary Data are available at NAR Online: Supplementary Figures 1–6, Supplementary Methods and Supplementary References [49–52].

## FUNDING

National Institutes of Health (NIH) [GM GM079154 to M.E.A.C. and W.H.L. by training grant T32GM007635]. Funding for open access charge: NIH.

*Conflict of interest statement*. None declared.

## Supplementary Material

Supplementary Data
