# CAF-1-induced oligomerization of histones H3/H4 and mutually exclusive interactions with Asf1 guide H3/H4 transitions among histone chaperones and DNA

**DOI:** 10.1093/nar/gkx729

**Published:** 2017-08-18

**Authors:** Wallace H. Liu, Sarah C. Roemer, Alex M. Port, Mair E.A. Churchill

**Affiliations:** Department of Pharmacology, University of Colorado School of Medicine, Mail Stop 8303, PO Box 6511, Aurora, CO 80045, USA

*Nucleic Acids Res* (2012) 40 (22): 11229–11239. doi :10.1093/nar/gks906

The authors wish to make the following correction to their article:

The parts of the model presented in Figure 6 illustrating the stoichiometry of the CAF-1:H3/H4 complex as 1:2 are not consistent with recent published literature, which report both a 1:1 or 2:2 stoichiometry under different sets of conditions (1,2).

All of the experiments and results in this publication (Liu *et al.*, 2012) are reproducible in our hands. All of the other conclusions of the study and all of the other aspects of the model stand as published. The description of the data that led to the conclusion of the 1:2 CAF-1:H3/H4 stoichiometry is presented:

**Figure 4A, D and Figure S4 led to the conclusion that** CAF-1 associates with multimers of H3/H4 in the absence of DNA. We now believe that the mixed fluorophore FRET assay interpreted to form this conclusion is not reliable for proving H3/H4 tetramerization by CAF-1. Despite the reproducibility of this assay and the concordance of the control experiments in Figures 4B, 4C, and S3 with the established literature, the assay appears to give an erroneous FRET effect for the CAF-1-H3/H4 interaction for unknown reason(s). This fluorescence assay can be used to report on binding affinities and gives reliable KD values for H3/H4 with CAF-1, but it does not report the proximity between H3/H4 dimers. We suspect that the spectral changes that occur with CAF-1 are a result of fluorophore quenching and/or weak photostability. As such, we caution against the use of this approach in Figure 4A to study histone oligomerization.

**Figure 6:** The CAF-1:H3/H4 stoichiometry: The parts of the model presented in this figure illustrating the CAF-1:H3/H4 stoichiometry to be 1:2 are not consistent with recent published literature (Mattiroli *et al.*, 2017, Sauer *et al.*, 2017). All other aspects of the model stand.
