# Supplementary material for: CAF-1-induced oligomerization of histones H3/H4 and mutually exclusive interactions with Asf1 guide H3/H4 transitions among histone chaperones and DNA
Source: Nucleic Acids Res. 2012 Oct 2;40(22):11229–39. doi: 10.1093/nar/gks906 (PMC3526290; doi:10.1093/nar/gks906)
Supplement: Supplementary Data [file supp_40_22_11229__index.html]

CAF-1-induced oligomerization of histones H3/H4 and mutually exclusive interactions with Asf1 guide H3/H4 transitions among histone chaperones and DNA — CAF-1-induced oligomerization of histones H3/H4 and mutually exclusive interactions with Asf1 guide H3/H4 transitions among histone chaperones and DNA — Supplementary Data 

# CAF-1-induced oligomerization of histones H3/H4 and mutually exclusive interactions with Asf1 guide H3/H4 transitions among histone chaperones and DNA

## Supplementary Data

files

**Files in this Data Supplement:**

- Supplementary Data - docx file
