# Supplementary material for: CAF-1-induced oligomerization of histones H3/H4 and mutually exclusive interactions with Asf1 guide H3/H4 transitions among histone chaperones and DNA
Source: Nucleic Acids Res. 2012 Oct 2;40(22):11229–39. doi: 10.1093/nar/gks906 (PMC3526290; doi:10.1093/nar/gks906)
Supplement: Supplementary Data [file supp_gks906_nar-02014-f-2012-File008.docx]

Supplementary Methods.

Supplementary Figures S1-S6.

Supplementary References [49-52].

**CAF-1-induced oligomerization of histones H3/H4 and mutually exclusive interactions with Asf1 guide H3/H4 transitions among histone chaperones and DNA**

Wallace H. Liu, Sarah C. Roemer, Alex M. Port, and Mair E.A. Churchill*

Department of Pharmacology, University of Colorado School of Medicine, Mail Stop 8303, PO Box 6511, Aurora CO 80045, USA

* To whom correspondence should be addressed:

phone: 303-724-3670

fax 303-724-3663

email: [mair.churchill@ucdenver.edu](mailto:mair.churchill@uchsc.edu)

**Supplementary Methods**

**Preparation of Expression Vectors**

Site-directed mutagenesis using the Quikchange II XL kit (Stratagene) was used to introduce a single point glutamate mutation in H3.

Cac3 was sub-cloned by a single step polymerase chain reaction (PCR) and Cac1-StrepII and Cac2-His_6_ by a two-step PCR amplification from yeast gDNA, then inserted into the Gateway pDONR/Zeo vector (Invitrogen) by recombination. For the two step PCR, the first amplification primers included the N-terminal AttB1 site required for recombination into the pDONR/Zeo vector, while the antisense primers included the sequence for the respective C-terminal tags for Cac1-StrepII and Cac2- His_6_. The same sense primers were used for the second step of amplification along with antisense primers containing the C-terminal AttB2 sequence required for recombination into the pDONR/Zeo vector. Correct recombination was confirmed by dideoxy sequencing. The primer sequences used were:

Cac1 sense: 5’GGGGACAAGTTTGTACAAAAAAGCAGGCTAAATGGAGCAACATCTCAAATCAATTC3’

Cac1 antisense #1: 5’CTACTTTTCGAACTGCGGGTGGCTCCATCCTGACAAAGACGGGGTTGGCATATT3’

Cac1 antisense #2: 5’GGGGACCACTTTGTACAAGAAAGCTGGGTTTACTACTTTTCGAACTGCGGGTG3’

Cac2 sense: 5’GGGGACAAGTTTGTACAAAAAAGCAGGCTCAATGGAAGCTTCACATTTGCAAA3’

Cac2 antisense #1: 5’CTATTAGTGGTGATGGTGATGGTGTCCTGACAAATCGACTGGCGTAGGAT3’

Cac2 antisense #2: 5’GGGGACCACTTTGTACAAGAAAGCTGGGTCTATTAGTGGTGATGGTGATGG 3’

Cac3 sense: 5’GGGGACAAGTTTGTACAAAAAAGCAGGCTAGATGAATCAGTGCGCGAAGG3’

Cac3 antisense: 5’GGGGACCACTTTGTACAAGAAAGCTGGGTCTATCACGAATGTCCAACAAGGTTTC 3’

To produce baculovirus transfer vectors, protein sequences were amplified from the pDONR/Zeo vectors via PCR. To add a FLAG tag to the Cac3 subunit a two-step PCR (as above) was used. PCR products were inserted into the baculovirus transfer vector pVL1392 (Orbigen) using Not1 and BamH1 cloning sites. The pVL1392 transfer vectors were confirmed to be correct by dideoxy sequencing. Primer sequences used were:

Cac1 sense: 5’GCATGCGGCCGCATGGAGCAACATCTCAAATCAATTC3’

Cac1 antisense: 5’CGTAGGATCCTTACTACTTTTCGAACTGCGGG3’

Cac2 sense: 5’GCATGCGGCCGCATGGAAGCTTCACATTTGCAAATC3’

Cac2 antisense: 5’CGTAGGATCCCTATTAGTGGTGATGGTGATGG3’

Cac3 sense: 5’GCATGCGGCCGCATGAATCAGTGCGCGAAGGAC3’

Cac3 antisense #1: 5’TTACTACTTATCGTCGTCATCCTTGTAATCCGAATGTCCAACAAGGTTTCC

Cac3 antisense #2: 5’CGTAGGATCCTTACTACTTATCGTCGTCATCCTTG3’

The B domain truncation of Cac2 was generated by a two-step PCR amplification of aa 14 to 444 from the full length pDONR/Zeo Cac2-His_6_ vector using the aa14 sense primer: 5’GGGGACAAGTTTGTACAAAAAAGCAGGCTCAATGCAACCCGTTTACTCGCTCAC3' and antisense #1 5’CTATTAGTGGTGATGGTGATGGTGTCCTGACAGCATGTTCACGATCCCAC 3’ and antisense #2 5’GGGGACCACTTTGTACAAGAAAGCTGGGTCTATTAGTGGTGATGGTGATGG 3’.

The bacterial expression vectors for Cac2-His_6_ and Cac2 lacking the B domain were generated by recombination of the pDONR/Zeo p60-His_6_ vectors with pDEST-550 (NIH).

**
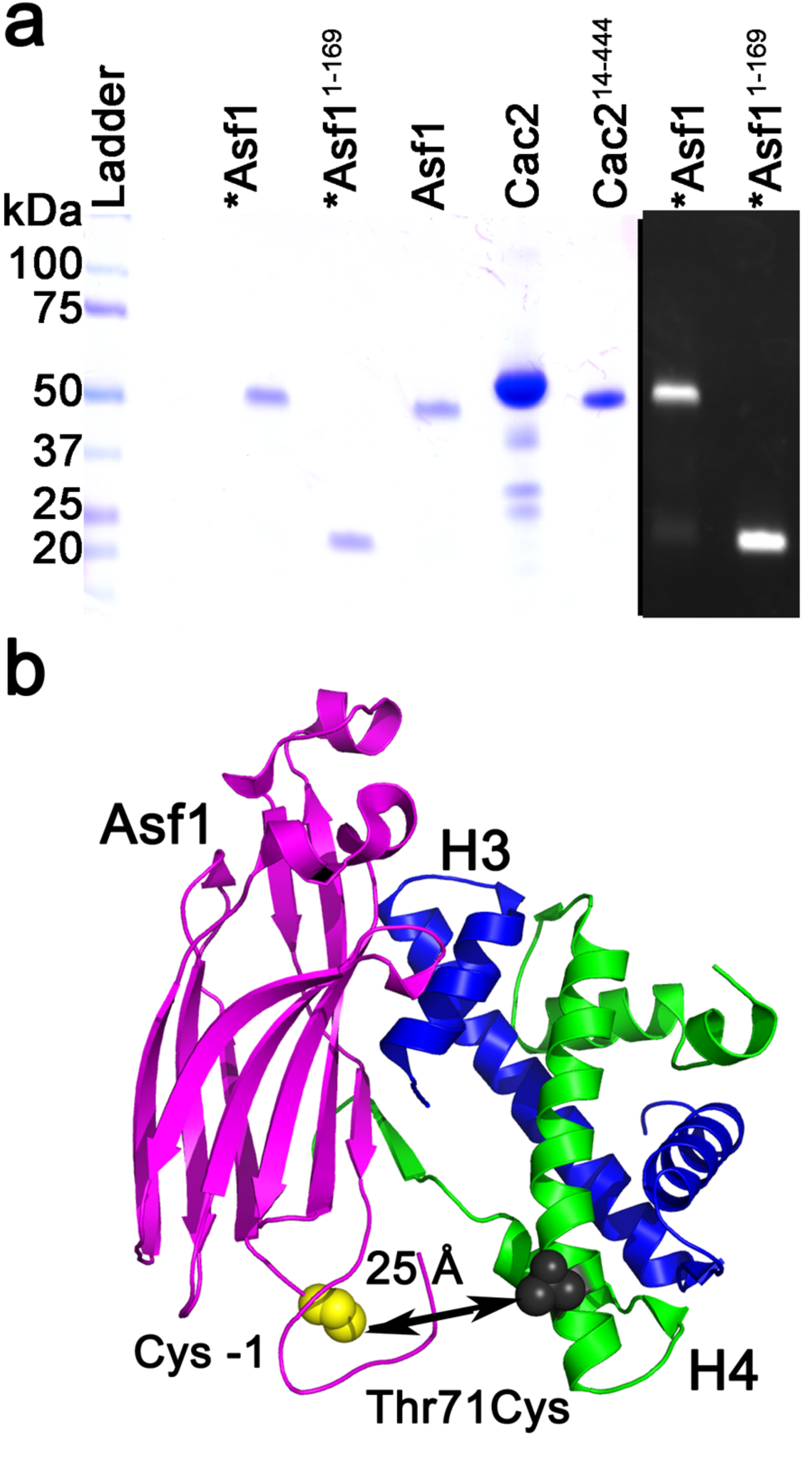
Figure S1.**

**Figure S1. Asf1 and Cac2 proteins and labeling schemes used in this study. a)** SDS-PAGE of purified proteins. The same gel was excited with UV light (right), then Coomassie blue stained (left). **b)** Schematic showing positions of *Asf1 labeled at the -1 Cysteine position (residue added at position -1) with Alexa Fluor 532, and H3/H4 labeled at cysteine 71 of H4 with fluorescein.

**
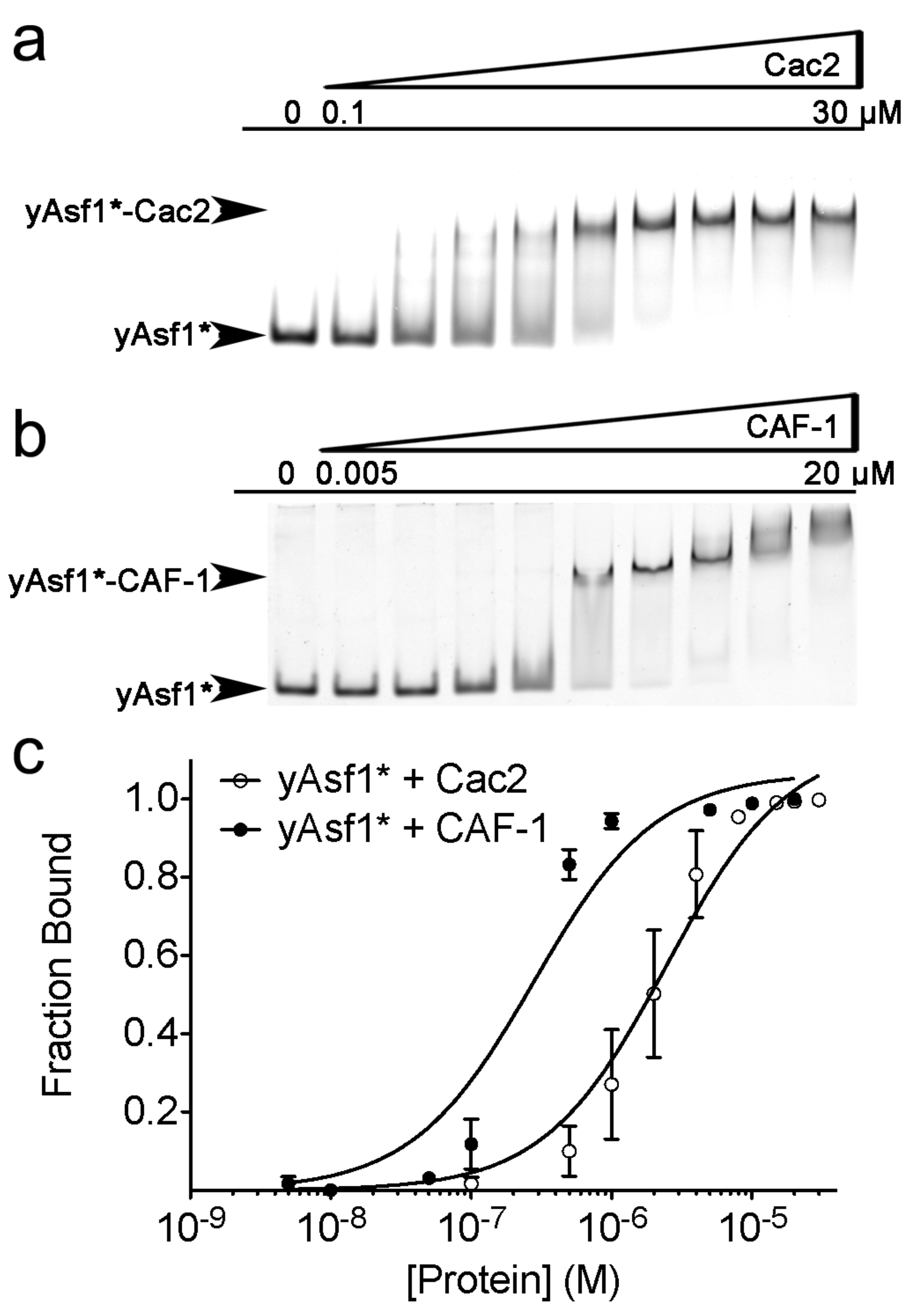
 Figure S2.**

**Figure S2. Asf1 binds more tightly to intact CAF-1 than to the Cac2 subunit alone.**  EMSA showing 100 nM labeled Asf1 bound to increasing concentrations of **a)** Cac2 and **b)** CAF-1 complex. *Asf1 species were detected on a Typhoon 9400 imager. **c)** The fraction of bound *Asf1 species were quantified, and then plotted against the concentration of Cac2 or CAF-1.

**Figure S3.**


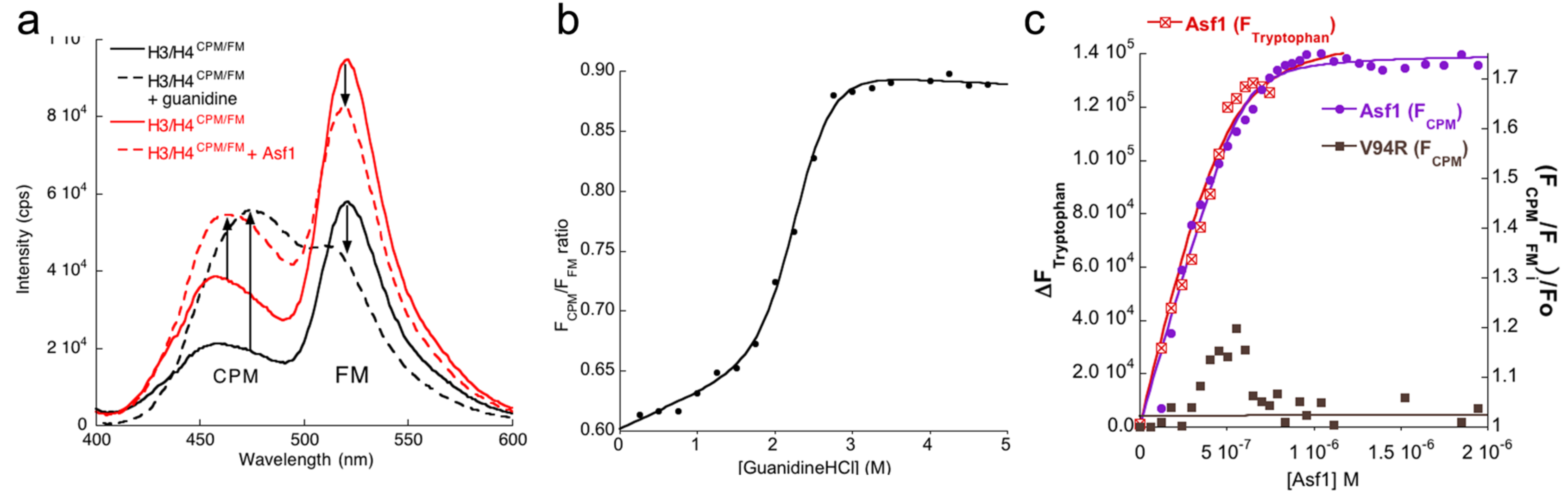


**Figure S3. Analysis of fluorophore-labeled H3/H4 under conditions where the histones adopt a tetrameric configuration.** **a)** H3/ H4^CPM/FM^ FRET is decreased in 3M guanidine hydrochloride (black) or by addition of stoichiometric amounts of Asf1 (red). The H3/H4^CPM/FM^ (500 nM) complexes were excited at 385 nm in buffer (solid black line) or in 3 M guanidine HCl buffer (dotted black line). Increased CPM emission and concomitant decreased FM emission show a FRET signal that decreases upon addition of Asf1 or guanidine. **b)** Guanidine dissociation curve of H3/H4 tetramers, fitted with a two-state dissociation model (49,[50](#_ENREF_52)): Y = ((y^°^_N_ + m_N_[Gua]) + (y^°^_U_ + m_U_[Gua])*exp[(-ΔG°_H2O_ + m[Gua])/RT])/ (1+exp[(‑ΔG°_H2O_+m[Gua])/ RT]), where y_N_ and y_U_ represent the fluorescence contribution from the native and unfolded states, respectively, at a specific denaturant concentration; R is the gas constant, T is temperature, y^°^_N_ and y^°^_U_ are the values of the fluorescence extrapolated to 0 M denaturant; and m_N_ and m_U_ are the slopes of the pre- and post-transition regions, respectively. **c)** H3/H4 tetramer dissociation and Asf1 binding curves. Asf1 or Asf1V94R were titrated into mixed-labeled H3/H4^CPM/FM^ tetramers. F_CPM_/F_FM_ is a measure of FRET ratio for each Asf1 titration point observed at one histone concentration (6 x 10^-7^ M). The fluorescence quenching of Asf1 tryptophan (F_tryptophan_) was also monitored, as the difference between the fluorescence at 340 nm of Asf1 titrated into buffer and Asf1 titrated into H3/H4. The curves were fitted with a ligand-depleted binding model, F_i_ = 1+(ΔF_max_)*(((K_D_+[H3H4]+[Asf1]_i_)-sqrt(((K_D_+[H3H4]+[Asf1]_i_)^2^)-(4*[H3H4]*[Asf1]_i_)))/ 2*[H3H4]), where [H3H4] is the histone dimer concentration and [Asf1] is the Asf1 concentration at each point ([51](#_ENREF_53)).


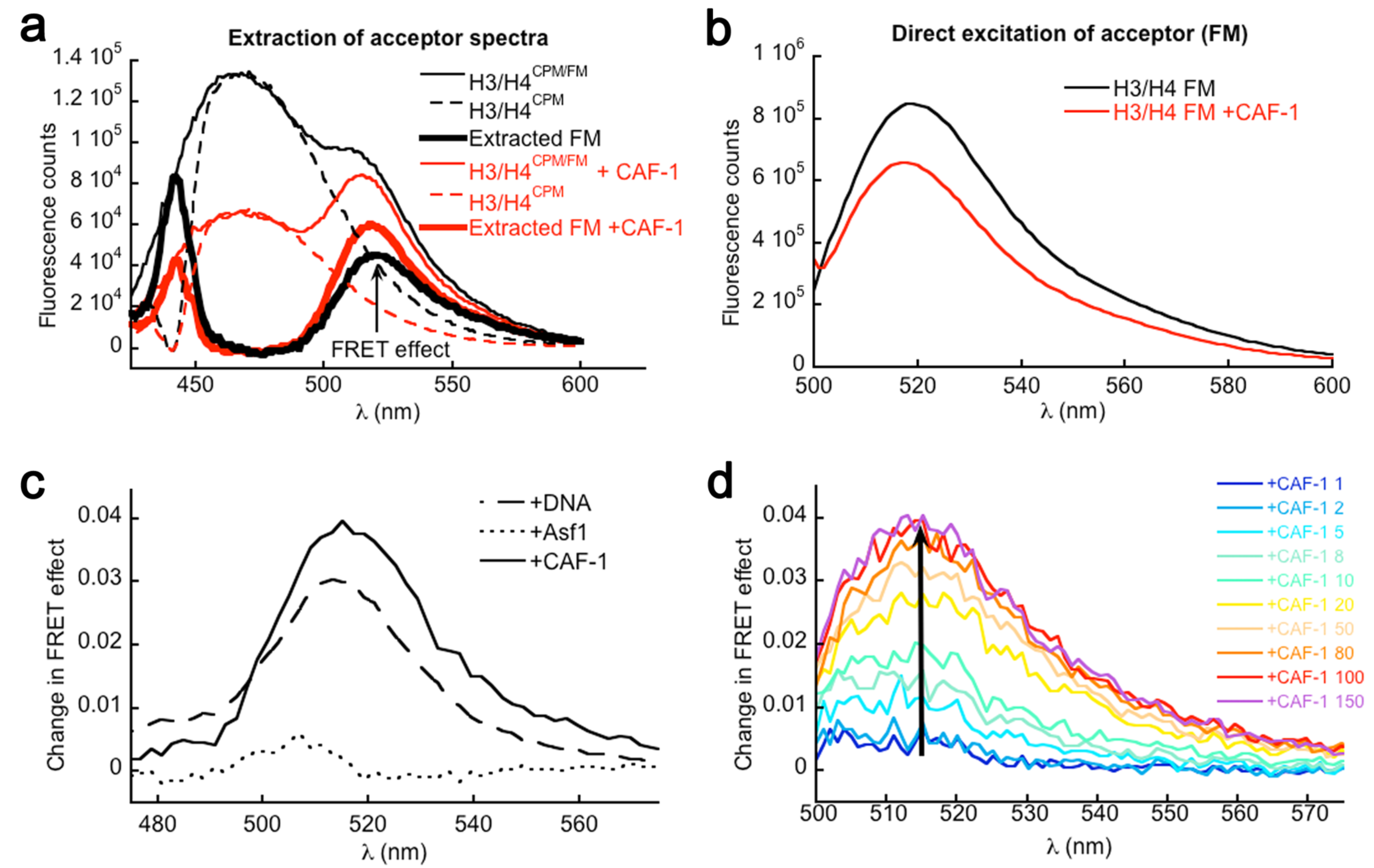
**Figure S4.**

**Figure S4. Fluorescence analysis of the CAF-1 interaction with labeled H3/H4.**  **a)** Fluorescence emission spectra of 2 nM CPM- and FM-labeled H3/H4 (H3/H4^CPM/FM^) (black line), and 2 nM H3/H4 ^CPM/FM^ with 100 nM CAF-1 (red line) excited at 385 nm. The spectra of donor fluorophore labeled (CPM) H3/H4 (H3/H4^CPM^) were fitted to these (black and red dotted lines) and subtracted from them to give the extracted spectra shown in heavy black and red lines. There is residual acceptor signal even in the absence of CAF-1, which is attributed to the small amount of direct excitation of the acceptor fluorophore (FM). **b)** CAF-1 quenches acceptor fluorescence. Direct excitation of the acceptor of 2 nM H3/H4^CPM/FM^ (black line), and 2 nM H3/H4^CPM/FM^ with 100 nM CAF-1 (red line) excited at 491 nm. **c)** DNA and CAF-1 but not Asf1 induce FRET in CPM/FM-labeled H3/H4. The change in FRET effect is shown for 10 nM H3/H4^CPM/FM^ in the presence of 100 nM CAF-1 (solid line), 100 nM 80 bp 601 DNA (dashed line), or 100 nM Asf1 (dotted line). The FRET effect was calculated by normalizing the extracted FRET spectra (as in panel **a**), with the observed acceptor quenching (panel **b**). The H3/H4 alone spectra were then subtracted to show the change in FRET effect. **d**) CAF-1 binding induces FRET in H3/H4^CPM/FM^. Plots of the change in FRET effect observed for 2 nM H3/H4^CPM/FM^ in the presence of increasing amounts of CAF-1 (calculated as described in panel **c)**).

**Figure S5.**

**
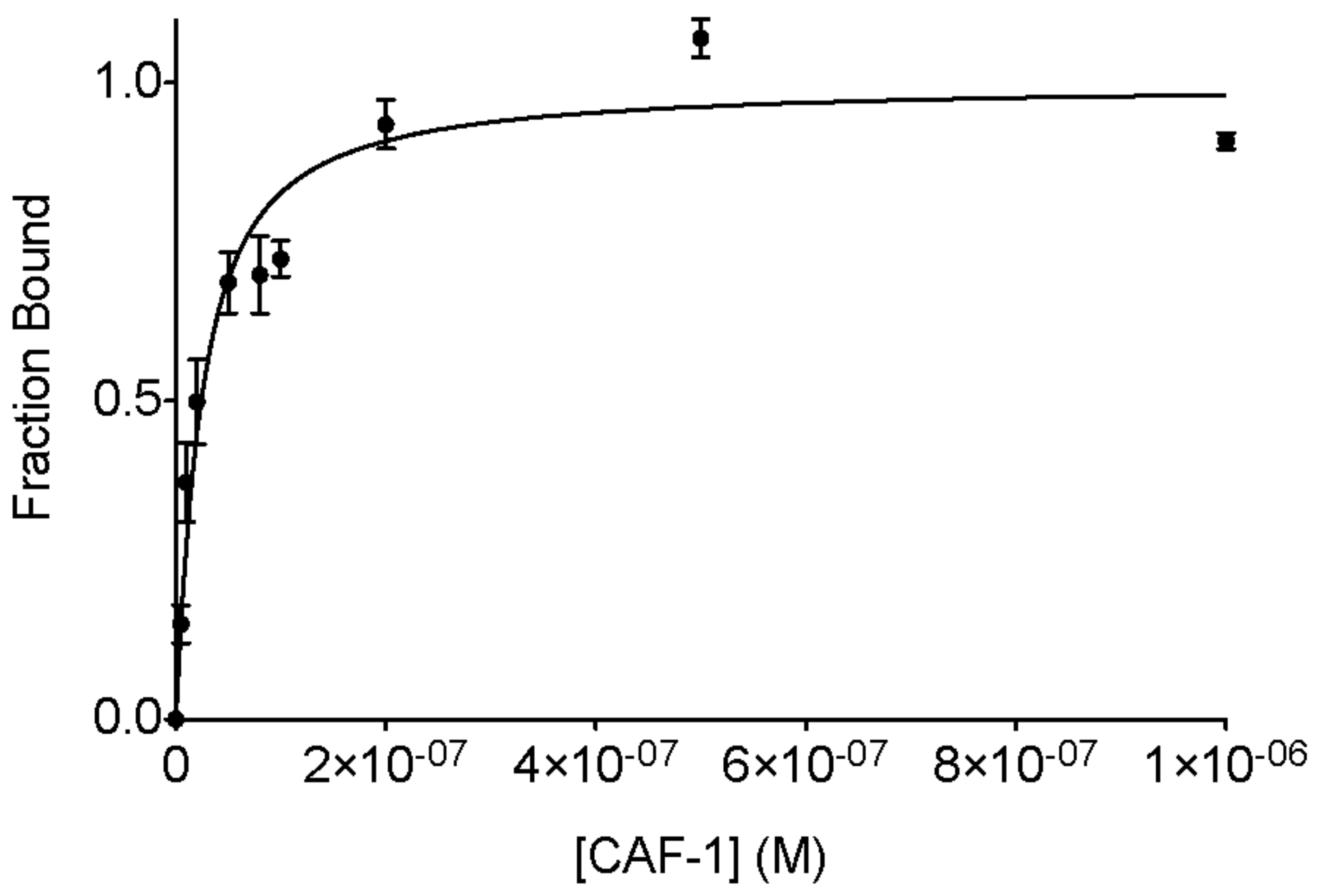
**

**Figure S5. K_D_ determination of the CAF-1 interaction with H3/H4 from EMSA analysis.**  10 nM of H3/H4 labeled with FM were bound in each well with concentrations of CAF-1 ranging from 0 – 1 µM in a 0.2x TBE native gel. The K_D_ of the interaction was determined by a ligand-depleted binding model: Y=((BMax)*((([H3H4]+[CAF-1]+K_D_)-sqrt((([H3H4]+[CAF-1]+K_D_)^2)-(4*[CAF-1]*[H3H4])))/(2*[H3H4]))) ([51](#_ENREF_53)).


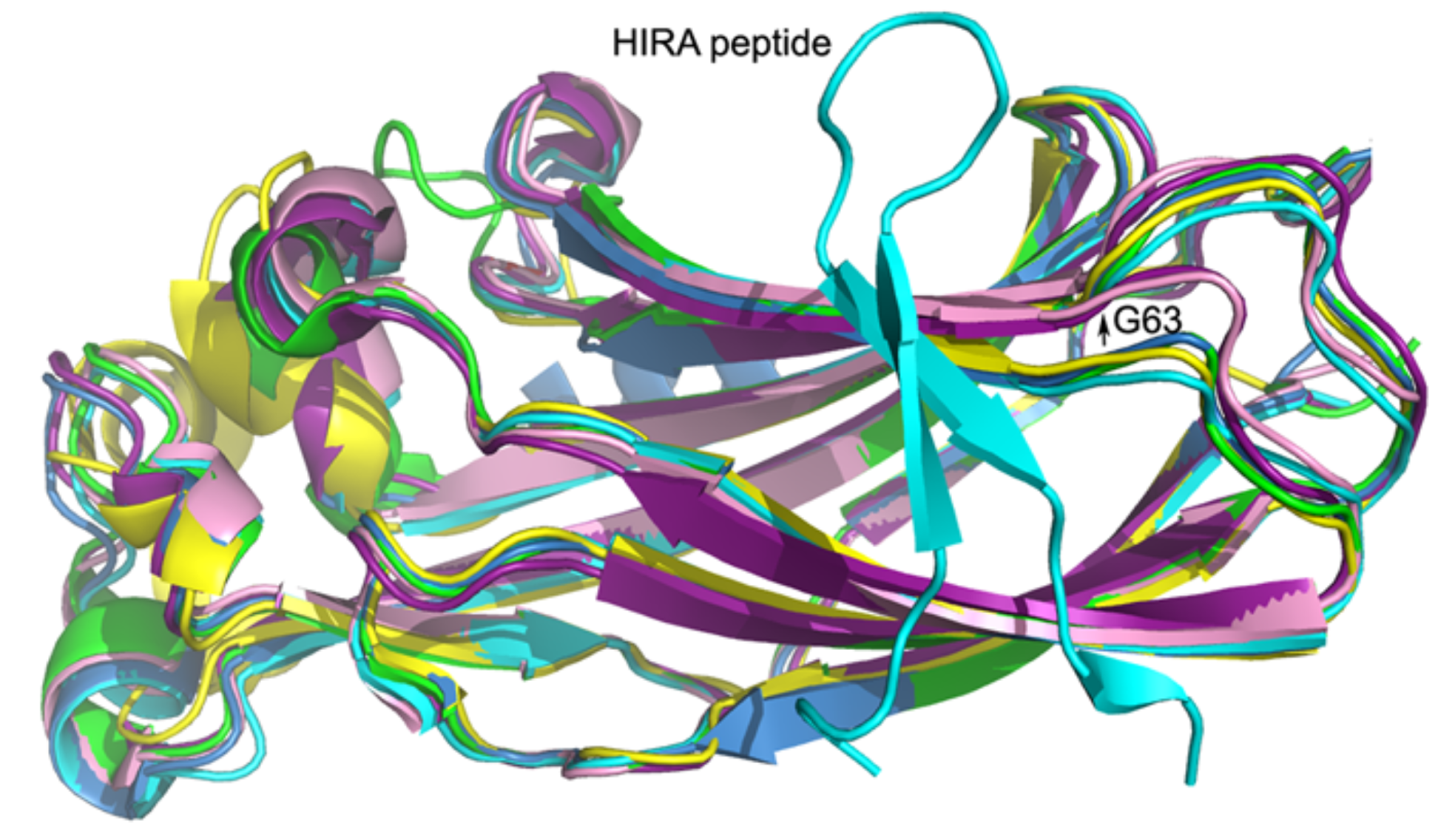
**Figure S6.**

**Figure S6. Asf1 structures aligned.** The superimposed structures are Asf1 free (PDB ID 1ROC ([41](#_ENREF_44)), green), Asf1 bound to an H3 peptide (PDB ID 2IDC ([52](#_ENREF_54)), blue), Asf1 bound to an H3 peptide (PDB ID 2DZE, yellow), Asf1 bound to a HIRA B-domain peptide (PDB ID 2I32 ([23](#_ENREF_23)), cyan), and Asf1 bound to H3/H4 (yeast: PDB ID 2HUE ([16](#_ENREF_16)), purple; human: PDB ID 2IO5 ([17](#_ENREF_17)), pink). The HIRA peptide is shown to indicate the location of its binding site. The histones bind on the top and opposite face and are not shown. However, only the H3/H4 bound structures show a structural shift in the beta-strand containing glycine 63, indicated by the black arrow. In this region there is a nearly 2 Å shift and twist in the beta sheet.

**References.**

49. Pace, C.N. Determination and analysis of urea and guanidine hydrochloride denaturation curves. *Methods Enzymol* **131**, 266-80 (1986).

50. Santoro, M.M. & Bolen, D.W. Unfolding free energy changes determined by the linear extrapolation method. 1. Unfolding of phenylmethanesulfonyl alpha-chymotrypsin using different denaturants. *Biochemistry* **27**, 8063-8 (1988).

51. Swillens, S. Interpretation of binding curves obtained with high receptor concentrations: practical aid for computer analysis. *Mol Pharmacol* **47**, 1197-203 (1995).

41. Daganzo, S.M. et al. Structure and function of the conserved core of histone deposition protein Asf1. *Curr Biol* **13**, 2148-58 (2003).

52. Antczak, A.J., Tsubota, T., Kaufman, P.D. & Berger, J.M. Structure of the yeast histone H3-ASF1 interaction: implications for chaperone mechanism, species-specific interactions, and epigenetics. *BMC Struct Biol* **6**, 26 (2006).

23. Tang, Y. et al. Structure of a human ASF1a-HIRA complex and insights into specificity of histone chaperone complex assembly. *Nat Struct Mol Biol* **13**, 921-9 (2006).

16. English, C.M., Adkins, M.W., Carson, J.J., Churchill, M.E. & Tyler, J.K. Structural basis for the histone chaperone activity of Asf1. *Cell* **127**, 495-508 (2006).

17. Natsume, R. et al. Structure and function of the histone chaperone CIA/ASF1 complexed with histones H3 and H4. *Nature* **446**, 338-41 (2007).
